# The Impact of Ultrashort Pulse Laser Structuring of Metals on In-Vitro Cell Adhesion of Keratinocytes

**DOI:** 10.3390/jfb15020034

**Published:** 2024-01-29

**Authors:** Susanne Staehlke, Tobias Barth, Matthias Muench, Joerg Schroeter, Robert Wendlandt, Paul Oldorf, Rigo Peters, Barbara Nebe, Arndt-Peter Schulz

**Affiliations:** 1 Institute for Cell Biology, University Medical Center Rostock, 18057 Rostock, Germany; barbara.nebe@med.uni-rostock.de; 2 Laboratory for Biomechanics, BG Hospital Hamburg, 21033 Hamburg, Germany; t.barth@bgk-hamburg.de (T.B.); m.muench@bgk-hamburg.de (M.M.); arndt-peter.schulz@imte.fraunhofer.de (A.-P.S.); 3 Clinic for Orthopedics and Trauma Surgery, University Hospital Schleswig-Holstein, Campus Lübeck, 23538 Lübeck, Germany; joerg.schroeter@uksh.de (J.S.); robert.wendlandt@uksh.de (R.W.); 4 SLV Mecklenburg-Vorpommern GmbH, 18069 Rostock, Germany; oldorf@slv-rostock.de (P.O.); peters@slv-rostock.de (R.P.); 5 Fraunhofer Research Institution for Individualized and Cell-Based Medical Engineering, 23562 Lübeck, Germany

**Keywords:** fracture, external fixator pin, laser structuring, wettability, keratinocytes, cell adhesion, spreading, actin cytoskeleton, antler

## Abstract

Besides the need for biomaterial surface modification to improve cellular attachment, laser-structuring is favorable for designing a new surface topography for external bone fixator pins or implants. The principle of this study was to observe how bioinspired (deer antler) laser-induced nano–microstructures influenced the adhesion and growth of skin cells. The goal was to create pins that allow the skin to attach to the biomaterial surface in a bacteria-proof manner. Therefore, typical fixator metals, steel, and titanium alloy were structured using ultrashort laser pulses, which resulted in periodical nano- and microstructures. Surface characteristics were investigated using a laser scanning microscope and static water contact angle measurements. In vitro studies with human HaCaT keratinocytes focused on cell adhesion, morphology, actin formation, and growth within 7 days. The study showed that surface functionalization influenced cell attachment, spreading, and proliferation. Micro-dimple clusters on polished bulk metals (DC20) will not hinder viability. Still, they will not promote the initial adhesion and spreading of HaCaTs. In contrast, additional nanostructuring with laser-induced periodic surface structures (LIPSS) promotes cell behavior. DC20 + LIPSS induced enhanced cell attachment with well-spread cell morphology. Thus, the bioinspired structures exhibited a benefit in initial cell adhesion. Laser surface functionalization opens up new possibilities for structuring, and is relevant to developing bioactive implants in regenerative medicine.

## 1. Introduction

The present study focuses on the optimization of external bone fixators in humans. External fixation is a versatile and minimally invasive method for treating various bone diseases, mainly fractures, non-unions, and arthrodesis of joints [1,2]. The fixator pins (also called Schanz Pins) are drilled into the affected bone of the patient via the skin [3,4,5,6]. They cross the human skin barrier during treatment lasting from a few days to several months [1,2]. Outside the human body, additional rods ensure that the position of the bone is held or that a malposition is successively corrected. Since this system is outside the human body, the Schanz Pins break through the skin barrier, which defines these as percutaneous implants. Because these titanium alloy or stainless steel pins are polished to achieve good cleanability, the skin cells do not adhere [2,3,4,5,6]. In the end, pathogen entry points remain at the skin level during treatment [1,3,7]. Although external fixators offer several advantages over more invasive therapies, various complications often occur during external fixator therapy [1,2,3,4]. Infection around the external fixator pins is the most common and significant complication, often requiring the revision and changing of treatment form. Incidences of infection ranging from 16 to 100% have been reported in the literature [1,3,4,7,8]. Therefore, researchers have searched for an appropriate surface modification method to reduce bacterial ingrowth for a long time [3,4,9,10,11].

**Figure 1:**
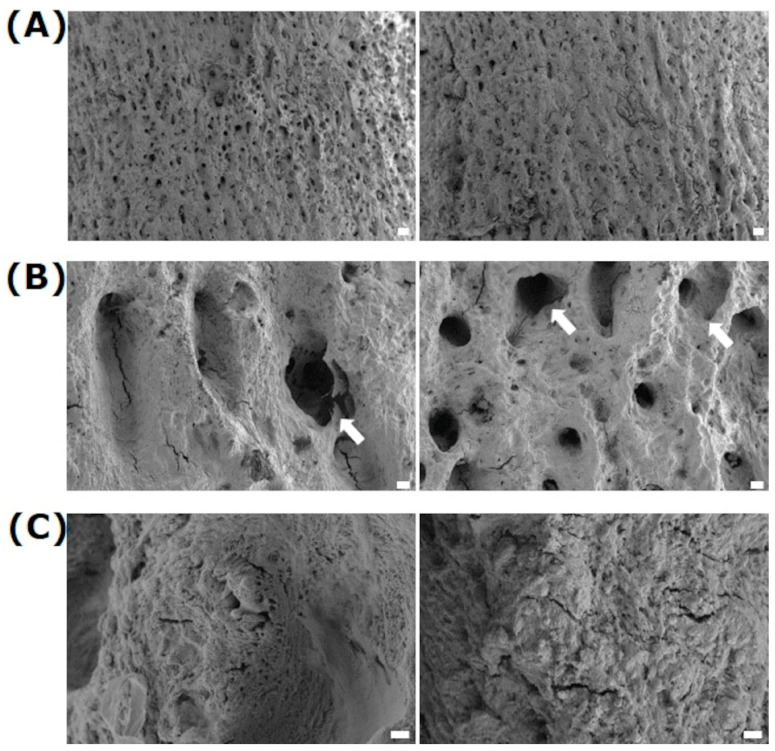
Exemplary images of deer antlers: (**A**) overview, (**B**) with their specific microstructures, and (**C**) with their nanostructures within the microstructures. Note that ~26 micro-cavities (arrows) per mm^2^ (26.2 ± 1.9) could be observed with a dimension of 41.9 ± 2.4 µm, mean ± s.e.m. (FE-SEM Merlin VP compact, 5 kV, HE-SE detector; scale bars: (**A**) = 100 µm, (**B**) = 20 µm, (**C**) = 2 µm)).

Also, in other medical areas where metallic percutaneous implants breach the skin barrier, such as dental implants, surface alterations have been a field of research and development for a while [12,13]. The innovative idea is to structure pins to allow skin cells to adhere to metallic structures and thus eliminate the opening for bacterial colonization. A model was found in the animal world—deer antlers—to overcome the high infection rates and implement the idea [14]. In deer, the antlers always grow out of the skin without causing inflammation: the antlers’ rough surface in the pedicle’s essential area allows the skin to adhere securely and close the skin barrier [15]. Scanning electron microscopy (SEM) images of the surface of the antler showed specific nano- and microstructures (Figure 1). It should now be clarified whether the bioinspired structuring can be transferred to metal and promote the growth of skin cells. To the best of our knowledge, this is the first study regarding the adhesion behavior of keratinocytes on laser-structured metal surfaces.

The interaction of human cells with implant surfaces is critical and contributes to clinical success [16]. Cells can sense their physico-chemical environment, triggering the reorganization of focal adhesion, the spatiotemporal activation of signaling molecules like calcium ion mobilization, and phenotypic changes, e.g., of the actin cytoskeleton [17]. The adhesion of cells to the surrounding extracellular matrix is the basis for developing and maintaining a functioning biosystem [18]. Adhesion proteins, such as integrins, link the extracellular matrix molecules and the intracellular actin cytoskeleton via focal adhesion. As a result, they regulate numerous physiological processes [19]. Cells can translate external signals and forces from the extracellular environment at the interface to implant surfaces by converting physical stimuli into biochemical signals [17]. Physical stimuli include surface chemistry, topography, and elasticity [16]. It is known that nano- and microstructures influence cell behavior, such as adhesion, changes in the cytoskeleton, migration, growth, and differentiation [13,16,17,20,21]. The modification and optimization of the surface properties of implants with a focus on cell adhesion sites have been investigated by many research groups [16,21]. Surface characteristics like roughness and wettability influence cellular processes like spreading and proliferation [16,17,18,19,20,21,22]. However, especially on laser-structured metallic surfaces, a substantial change in the wetting state over time is observed [23,24], and the correlation between hydrophilicity and pronounced cell adhesion is questioned [25].

A key challenge for materials science is preparing biomaterials that promote tissue regeneration. The cell occupancy of a biomaterial’s surface is divided into different phases, starting with the initial attachment of cells, their spreading and growth, and cell differentiation [16]. We have previously shown that topographies with sharp edges alter the overall cell morphology and intracellular actin cytoskeleton formation [18], and affect extracellular matrix synthesis on the protein and mRNA levels [26]. Thus, edges, ridges, and spikes on titanium surfaces cause enhanced stress in osteoblasts and an increase in reactive oxygen species (ROS) in cells [26]. In addition, cell signaling was hampered by the slower mobilization of calcium ions from intracellular stores [17].

Gristina et al. [27] proposed the concept of a “race for the surface”, whereby host and bacterial cells compete in determining the ultimate fate of the metallic implant. It is now commonly accepted that the measure taken to reduce the bacterial burden on metallic implants has to be swift to allow a host response before biofilm formation occurs—in the best case, by the direct contact of host cells with the implant [28].

The transfer of such bioinspired “natural” nano- and microstructures is possible with ultrashort laser pulses (ultrafast lasers) on metals (steel and titanium alloy) [29,30]. Until now, however, the production of well-defined structures on this scale has not been possible, so attempts at etching or other randomized methods in wound healing have been unsuccessful [31,32,33]. As with conventional laser types, ultrafast lasers can be used for the drilling, cutting, structuring, and surface functionalization of workpieces. However, ultrashort laser pulses have significant advantages over other laser types; they can produce laterally microscopic structure with sizes down to a few micrometers. Due to the pulse durations in the femto- to picosecond range and the associated ultrashort light–matter interaction times, with simultaneously very high pulse peak powers, material removal takes place primarily in the vapor phase (sublimation), which leads to minimal heat input and thus negligible thermal stress on the workpiece. These facts make the ultrafast lasers a purely photonic, contamination-free, and exact tool.

The project aims to improve the main limitations of an established procedure for minimally invasive fracture treatment—the usage of an external fixator—using a naturally inspired solution. In addition to studies on the mechanical properties of materials after surface treatment with an ultrafast laser (biomechanical properties), in vitro studies were performed to test whether human skin cells adhere and grow better. Classically, most in vitro studies of cell response to biomaterials focus on either cell attachment after a few hours of contact or proliferation during a few days of culture.

In the current study, we will analyze the effects of different nano- and microstructures on the initial adhesion (10 min), morphology, and growth of human keratinocytes for up to 7 days.

## 2. Materials and Methods

### 2.1. Materials and Sample Pretreatment

The experimental investigations were carried out using medical steel (Stryker, Tuttlingen, Germany; stainless steel; No.: 1.4441, ASTM-F138, DIN EN ISO 5832-1:2019-12) and a titanium (Ti) alloy with alumina (Al) and vanadium (V) (Ti_6_Al_4_V, Grade 5; Stryker; ASTM F1472, DIN EN ISO 5832-3:2022-02). The disc-shaped samples had a diameter of 6 mm and a thickness of 1 mm. Before the rough steel samples, which were produced by electrical discharge machines (EDM, MV2400S, Mitsubishi Electric, Tokyo, Japan) from Schanz Pins, could be structured, they were embedded in epoxy resin and polished in an automatic surface grinding machine (LaboPol-30, Struers GmbH, Willich, Germany) to a roughness of arithmetical mean height (Sa) = 0.03 µm. After the samples were separated again, they were cleaned in an ultrasonic bath (Elmasonic P30H, Elma Schmidbauer GmbH, Singen, Germany) and with clean compressed air. The Ti_6_Al_4_V samples were not additionally polished before structuring and showed a finely turned surface with a roughness of Sa = 0.23 µm. The roughness measurements were performed according to ISO 25178 in a laser scanning microscope (VK-X200, Keyence, Osaka, Japan).

### 2.2. Micro-Structuring Using Ultrashort Laser Pulses

For the process development and laser structuring of the samples, a femtosecond laser of the type TruMicro 6020 (Trumpf GmbH & Co., KG, Ditzingen, Germany) with a wavelength of λ = 1030 nm, a repetition rate of 1 MHz, and a pulse duration of 530 fs was used [34]. The laser system was integrated into a precise 5-axis micromachining system of GL.evo (GFH GmbH, Deggendorf, Germany). Accurate beam guidance on the planar specimen geometries was carried out using an ExcelliSCAN 14 scanner system (Scanlab GmbH, Puchheim, Germany) with variable f-Theta lenses and corresponding different focal lengths. The laser-induced periodic surface structures (LIPSS) were nanostructured using surface hatching with linearly polarized laser pulses. In contrast, low fluences between 2.8 and 5.6 J/cm^2^ were used for the fine and highly quantitative structuring of various micro-dimple clusters (DC) without throw-ups and ridges. The fast structuring of the dimples was achieved using on-the-fly processes.

The task was to develop various combinations of dimple patterns and LIPSS in order to assess the deer antler’s porous structure as a bionic specification. Thus, ten different laser structures with n = 15 were transferred to the medical steel and Ti_6_Al_4_V samples after the process had been developed. In Appendix A, an overview of all investigated laser structures is summarized. For this study, we focused on micro-dimple clusters with a dimple dimeter of 20 µm (DC20), periodic nanostructures (LIPSS), and a combination of both micro-dimples and nanostructures (DC20 + LIPSS), which were compared to polished metals (Ref; steel and Ti_6_Al_4_V) (Table 1).

### 2.3. Surface Characterization

The laser-induced microstructures were characterized using a scanning electron microscope (SEM, EVO 15, Carl Zeiss Microscopy GmbH, Oberkochen, Germany) and a laser scanning microscope (VK-X200, Keyence, Osaka, Japan) [35] by measuring the dimple dimensions in their profile and determining the texture density (Table 1). Furthermore, after cleaning in an ultrasonic bath with deionized water and the subsequent drying of the surfaces, the structures were examined for their water contact angle in a wetting test rig of the type OCA11 (Dataphysics GmbH, Filderstadt, Germany). Here, the sessile drop method was used to determine the water contact angle using deionized water drops with a volume of 1 μL. Each contact angle measurement resulted in two wetting angles on the left and right sides of the droplet profile, from which the mean value was then calculated.

### 2.4. Cell Culture

In the in vitro studies focused on cell adhesion, morphology, and growth on laser-structured surfaces, human keratinocytes (HaCaT, CLS, Cell Lines Service GmbH, Eppelheim, Germany #330493) were used. HaCaTs are spontaneously transformed keratinocytes from histologically normal adult skin. These cells are used extensively to study epidermal homeostasis and its pathophysiology [36].

Cells were serially passaged at 70–80% confluence, followed by experiments with subconfluent cells. For this, HaCaTs were cultured in Dulbecco’s modified eagle’s medium (DMEM; high glucose, GlutaMAX-; Thermo Fisher Scientific, Gibco, Paisley, UK) containing 10% fetal bovine serum (FBS; Biochrom FBS Superior, EU-approved, Merck KgaG, Darmstadt, Germany) and 1% penicillin/streptomycin (Pen Strep; Thermo Fisher Scientific, Gibco, Paisley, UK) at 37 °C and in a 5% CO_2_ atmosphere (incubator, Sanyo CO_2_ incubator MCO-18AIC-UV, Panasonic Biomedical, Osaka, Japan). To detach the cells, for specific analyses, trypsin/ethylenediaminetetraacetic acid (0.25% trypsin/0.38% EDTA; Invitrogen, Gibco, Paisley, UK) was incubated at 37 °C for 7 min.

### 2.5. Cell Adhesion

The cell adhesion processes includes an essential in vitro parameter since material properties such as topography can thus be recorded directly as initial reactions at the material interface. At best, cells are already optimally adhered to the material after 10 min, although they have not yet spread. The 6 mm-diameter samples fitted optimally into one well of the 96-well plate. The number of cells was acquired using an FACSCalibur flow cytometer (BD Biosciences, Franklin Lakes, NJ, USA), equipped with a 488 nm argon laser. The software CellQuest Pro 4.0.1 (BD Biosciences, Franklin Lakes, NJ, USA) was used for data acquisition. For adhesion analysis via flow cytometry, 2 × 10^6^ cells/mL in suspension were stored at 37 °C and kept swinging until analysis. For the adhesion measurement, 100 µL of cell suspension was pipetted onto the surface. After the incubation of HaCaT cells at 37 °C for 10 min, the supernatant (containing non-adherent cells) was removed and pipetted into an FACS tube containing 200 µL of the medium. Then, the number of cells was analyzed on the FCASCalibur. As a blank, 100 µL of HaCaT suspension was pipetted into an FACS tube filled with 200 µL, and the total number of cells was first determined on the FACSCalibur (4 independent experiments).

Calculation:

Number of adherent cells = total number of cells in suspension − number of non-adherent cells

### 2.6. Morphology and Spreading of Keratinocytes

The morphology of HaCaTs (7 × 10^5^ cells for 2 h; 5 × 10^5^ for 1 d; 3 × 10^5^ cells for 7 d) was assessed using a field emission scanning electron microscope (FE-SEM, Merlin VP compact, Carl Zeiss, Oberkochen, Germany). For this, cells were washed after cultivation with N-(2-hydroxyethyl)-piperazine-N′-(2-ethane sulfonic acid) buffer (HEPES, Sigma-Aldrich, Munich, Germany), fixed with 2.5% glutardialdehyde (GA, Merck, Darmstadt, Germany) and dehydrated with an ascending ethanol concentration series (30% 5 min, 50% 5 min, 75% 10 min, 90% 15 min, 100% twice 10 min). Samples were dried in a critical point dryer (K850, Emitech, Taunusstein, Germany) and finally evaporated with carbon “C” under vacuum conditions (EM SCD 500, Co., Leica, Bensheim, Germany). An HE/SE (high-efficiency secondary electron detector) and an InlensDuo detector were used to image the cells (5 kV).

Using the FE-SEM images (500× magnification) of the 2 h HaCaT culture, the cell area of 40 cells per surface was calculated using ImageJ (National Institutes of Health, NIH) (n = 40 cells/specimens) [37].

### 2.7. Actin Cytoskeleton Organization

Actin filaments are dynamic structures essential to the cell’s shape, internal architecture, movement processes, and mass transport (signaling) [21,22]. The actin cytoskeleton organization of cells was determined using confocal laser scanning microscopy (cLSM; LSM780, Carl Zeiss Microscopy GmbH, Jena, Germany). Therefore, 5 × 10^5^ cells were cultured on the specimens for 24 h in a 48-well plate. After the 1-day incubation period, HaCaTs were washed three times with phosphate-buffered saline solution (PBS without Ca/Mg; Sigma-Aldrich, Munich, Germany). For fixation, the cells were stored in 4% paraformaldehyde (PFA; Sigma Aldrich) for 10 min, followed by washing steps with PBS (3×) and the permeabilization of the cell membrane with 0.1% 4-(1,1,3,3-Tetramethylbutyl) phenyl-polyethylene glycol (TRITON X-100; Merck KGaA) for 10 min. Finally, the cells were rewashed (3× PBS rinse). For actin staining, the cells were incubated with phalloidin-tetramethyl-rhodamine (TRITC, Sigma Aldrich; 1:15 in PBS) at RT in the dark for 1 h and embedded with Fluoroshield™ DAPI (Sigma Aldrich) after a wash step. Images of the actin cytoskeleton were taken using the LSM780 with software ZEN 2.3 (black edition) (Carl Zeiss) with the ZEISS oil immersion 63× objective (C-apochromat). To analyze the keratinocytes in the dimples, focused confocal images were acquired from multiple focal planes (Z-stack), which were merged into a 3D overlay for analysis [37].

### 2.8. Cell Viability

The cells’ growth, vitality, and thus viability were determined by MTS (3-(4, 5-dimethylthiazol-2-yl)-5-(3-carboxymethoxyphenyl)-2-(4-sulfophenyl)-2H-tetrazolium; CellTiter96^®^ AQueous One Solution Cell Proliferation Assay, Promega Corporation, Madison, WI, USA) and a crystal violet assay (Neisser solution II, Carl Roth GmbH + Co. KG, Karlsruhe, Germany). The MTS assay measures the activity of mitochondrial and cytosolic dehydrogenases (metabolic activity), and allows for the analysis of cytotoxicity and the initial control of cell response. Vital cells can convert MTS, a yellow tetrazolium, into soluble purple formazan. For this purpose, HaCaTs (7 × 10^5^ cells) were cultured in a 48-well plate (Thermo Fisher Scientific, Roskilde, Denmark). After a 22 h incubation period, the samples were transferred to a new 48-well plate to determine only the cells’ metabolic activity on the specimens. In the new plate, the MTS solution (1:6 in medium, 400 µL) was added and incubated for another 2 h at 37 °C. Finally, 100 µL of the supernatant was taken in three technical replicates and pipetted into a 96-well plate. The absorbance of the supernatant was eventually recorded at λ = 492 nm using a 96-well plate reader (ELISA Reader, Anthos 2010, Biochrom, Cambridge, UK). A background measurement was performed at 650 nm.

Crystal violet staining was performed to quantify the cell number of HaCaTs on specimens. Crystal violet binds linearly to negatively charged DNA by ionic attraction and detects the optical density of adherent growing cells. The staining was based on a previously published protocol [38]. Methodologically, after MTS measurement, adherent cells were washed three times with PBS, fixed with methanol, rewashed with 0.05% Tween20/PBS (3×; VWR Chemicals, Leuven, Belgium), and subsequently stained with a 0.1% Neisser reagent (Roth) for 20 min while shaking. After the staining step, the non-specific staining solution was washed out using double-distilled water, and the bound dye in the cells was extracted using acetic acid (33%; J. T. Baker, Deventer, Netherlands). Three samples of 100 µL of the supernatant, as technical replicates, were taken and the absorbance was measured in a 96-well plate in Anthos Reader at λ = 620 nm (4 independent experiments with three technical replicates each).

### 2.9. Statistical Analysis

All in vitro investigations were conducted at least three times in independent experiments. GraphPad Prism 7 (GraphPad Software Inc., La Jolla, CA, USA) was used to examine the statistical significance and prepare the graphs. Data are presented as mean ± standard error of the mean (s.e.m.). The group differences were considered by analysis of variance. At first, normal distribution (Shapiro–Wilk normality test) and homoscedasticity (Bartlett’s test for equal variances of k samples) were operated. According to the assumptions of the data, either a parametric one-way ANOVA with posthoc Bonferroni test and posthoc uncorrected Fisher’s LSD (for adhesion) or a nonparametric Kruskal–Wallis test post-tested with pairwise with Mann–Whitney U test using a Bonferroni-adjusted significance level (for cell spreading, and cell viability) was performed. The differences in *p*-values ≤ 0.05 (two-sided) were considered significant.

## 3. Results

### 3.1. Surface Characterization

Table 2 summarizes the samples’ averaged water contact angles for the differently structured medical steel and titanium alloy (Ti_6_Al_4_V). On polished medical steel, this resulted in an average water contact angle of θ = 59°. At the same time, the pure laser-induced periodic surface structure (LIPSS) led to a contact angle of θ = 67°. The micro-dimple cluster DC20 resulted in average contact angles of θ = 63° and, in combination with LIPSS, θ = 99°. Interestingly, all laser modifications on Ti_6_Al_4_V led to a decreased average water contact angle compared to medical steel (θ; LIPSS: 8.5°, DC20: 40.5°, DC20 + LIPSS: 28.2°).

**Figure 2:**
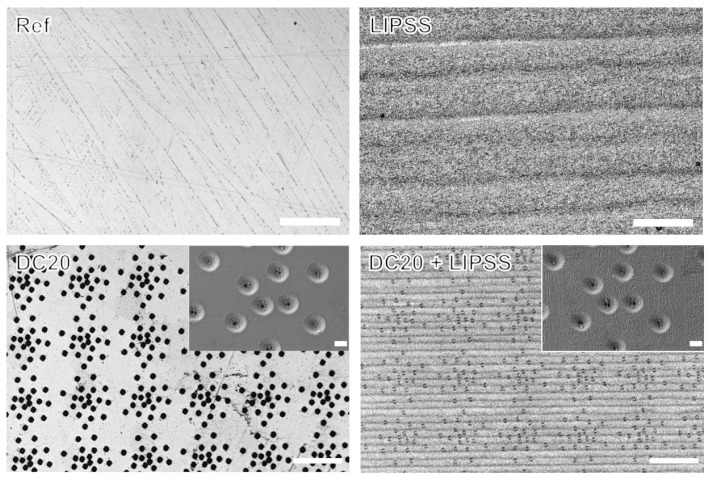
Exemplary images of medical steel surfaces: polished steel (Ref) as bulk surface, nanostructured LIPSS (polished surface with laser-induced periodic surface structure) (both: LSM, VK-X200, Keyence; scale bars = 50 µm), micro-dimple cluster DC20 (polished surface with 20 µm dimple diameter), and a combination of micro-dimples and nanostructures (DC20 + LIPSS) (Both: LSM, scale bars = 200 µm; inserts SEM EVO 15, Carl Zeiss; scale bars = 10 µm).

Figure 2 shows microscopic observations of the steel surfaces after polishing (Ref), those with nanostructured LIPSS, micro-dimple cluster DC20 featuring a dimple diameter of 20 µm at a density of 80 dimples per cluster, and the combination of micro-dimples and nanostructured DC20 + LIPSS. A similar characterization of the nano- and microstructures could also be achieved for titanium alloy. Details and images of all investigated laser-induced nano- and microstructures can be viewed in Appendix A.

### 3.2. Cell Response

The subsequent study focused on the effects of the laser-induced structuring of metals compared to polished surfaces (Ref) on the adhesion and growth of human keratinocytes (HaCaT) within the first 24 h up to 7 days. The results of all ultrafast laser modifications can be found in Appendix B.

#### 3.2.1. Cellular Adhesion

**Figure 3:**
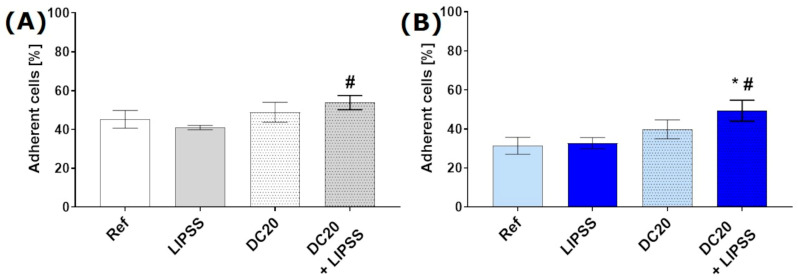
Initial adhesion of keratinocytes (HaCaT) on laser-structured specimens after 10 min: (**A**) on medical steel and (**B**) titanium alloy (Ti_6_Al_4_V). Note that the attachment of HaCaTs is significantly increased solely on the laser-induced micro-dimple cluster with additional nanostructures (DC20 + LIPSS) for both materials (FACSCalibur; n = 4 independent experiments, mean ± s.e.m., ordinary one-way ANOVA posthoc uncorrected Fisher’s LSD: * *p* ≤ 0.05 to Ref, # *p* ≤ 0.05 to LIPSS).

The initial phase of the cell–material interaction featured cellular attachment. Flow cytometry analysis for initial cell adhesion (10 min) was done to count the number of the non-adherent HaCaTs, followed by calculating the attached cells in % (Figure 3). A significant enhancement of adherent cells was detected on micro-dimple clusters with additional nanostructures (DC20 + LIPSS on medical steel: 54%, on Ti_6_Al_4_V: 49%) in comparison with the nanostructured specimens (LIPSS on medical steel: 41%, that on Ti_6_Al_4_V: 33%), and also on polished Ti_6_Al_4_V (Ref: 31%) (Figure 3). Interestingly, LIPSS showed no change in the attachment of HaCaTs compared to polished metals (Ref). The dimple micro-structured sample without LIPSS (DC20) indicated only a slight increase in initial adhesion (medical steel: 49%; Ti_6_Al_4_V: 40%).

#### 3.2.2. Cell Spreading

**Figure 4:**
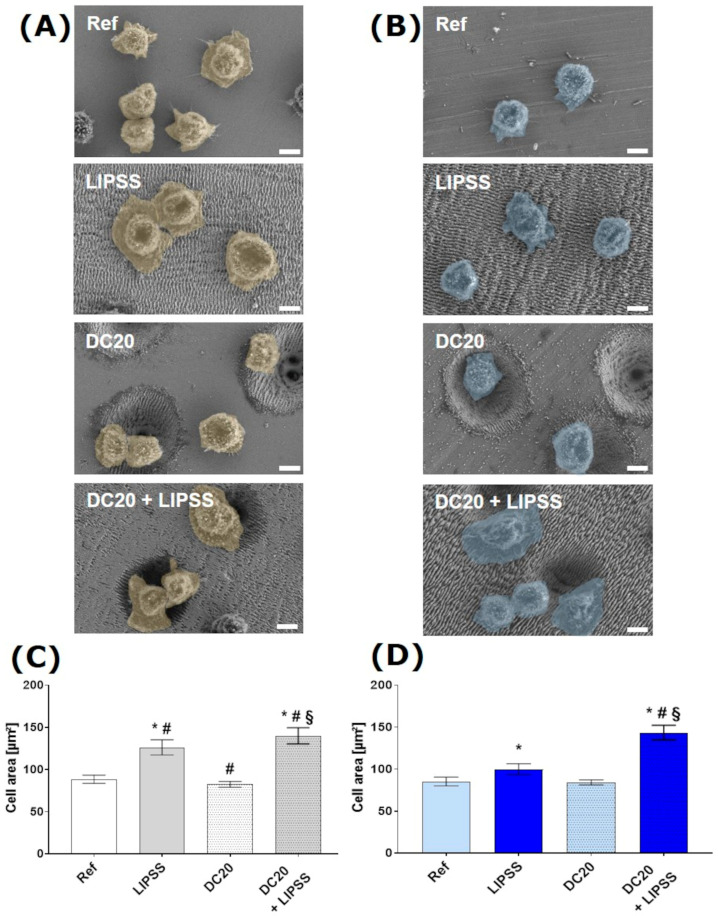
Cell spreading and growth of human keratinocytes (HaCaT) after 2 h on laser-structured metal specimens: (**A**) on medical steel and (**B**) on titanium alloy (Ti_6_Al_4_V). (FE-SEM Merlin VP compact, 2000× magnification, 5 kV, HE-SE detector, scale bars = 5 µm). The cell area of HaCaTs on (**C**) steel and (**D**) on Ti_6_Al_4_V. Note that the spreading of cells on both metals with micro-dimple clusters and additional nanostructures (DC20 + LIPSS) is significantly increased compared to all other specimens. Also, cells on the laser-induced periodic surface structures (LIPSS) shown on both materials achieved an enhanced cell area compared to polished metals (Ref). (ImageJ; n = 40 cells of SEM images, mean ± s.e.m., Kruskal–Wallis test post-tested with adjusted Mann–Whitney test: * *p* ≤ 0.05 to Ref, # *p* ≤ 0.05 to LIPSS, § *p* ≤ 0.05 to DC20).

The next stage of cell adhesion is the spreading of cells on the surface of a material, which means cells can flatten out. The laser-structured metal surfaces were analyzed to identify the most suitable structure for spreading as a sign of attachment and anchoring. Scanning electron microscopy (SEM) was used to monitor the spreading of HaCaTs on laser-structured medical steel (Figure 4A) and Ti_6_Al_4_V (Figure 4B). The SEM images of the keratinocyte’s 2 h spreading phase indicate that HaCaTs on LIPSS and DC + LIPSS spread well, and showed a more substantial development of the lamellipodia projection zone. In contrast, the HaCaTs on DC20 surfaces spread slowly, with a more compromised, rounded cell shape similar to those on polished metals (Ref).

The cell area was measured with ImageJ to calculate the spreading ability of HaCaTs. The spreading of HaCaTs after 2 h of cell cultivation on micro-dimple clusters with additional nanostructures DC20 + LIPSS achieved increased cell area compared with all other specimens (to Ref: medical steel ~1.6-fold, Ti_6_Al_4_V ~1.7-fold; to LIPSS: steel ~1.1-fold, Ti_6_Al_4_V ~1.4-fold; to DC20: steel ~1.7-fold, Ti_6_Al_4_V ~1.7-fold) (Figure 4C,D), regardless of the position of the cells on DC20 + LIPSS. Interestingly, while the initial adhesion was not affected, the nanostructured LIPSS shows increased cell area compared to polished material (to Ref: medical steel ~1.4-fold, Ti_6_Al_4_V ~1.2-fold). Meanwhile, the material with micro-dimple clusters DC20 indicated the same spreading as seen on polished materials (Ref) (Figure 4C,D).

#### 3.2.3. Cell Viability

**Figure 5:**
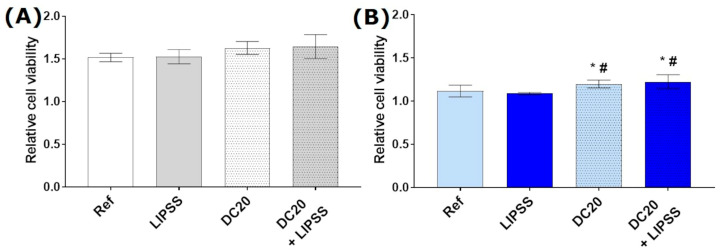
Relative cell viability of keratinocytes (HaCaT) on the different specimens after 24 h: (**A**) on steel and (**B**) on titanium alloy (Ti_6_Al_4_V). Cell metabolism (MTS assay) values were related to cell density (crystal violet). (Anthos reader; n = 4, mean ± s.e.m., Kruskal–Wallis test post-tested with adjusted Mann–Whitney test: * *p* ≤ 0.05 to Ref, # *p* ≤ 0.05 to LIPSS).

The metabolic activity per cell shows the impact of laser structuring on the viability of HaCaT keratinocytes. The relative viability of HaCaT keratinocytes after 24 h displayed an influence on laser-induced micro-dimple structures (DC20 and DC20 + LIPSS) (Figure 5). While the viability of the cells on the medical steel samples could only be slightly improved due to the laser structuring, a significant increase in the viability on titanium alloy laser-induced micro-dimple structures was detectable (all~1.1-fold).

A positive effect on growth due to laser structuring could be demonstrated.

#### 3.2.4. Cell Morphology and Actin Cytoskeleton

**Figure 6:**
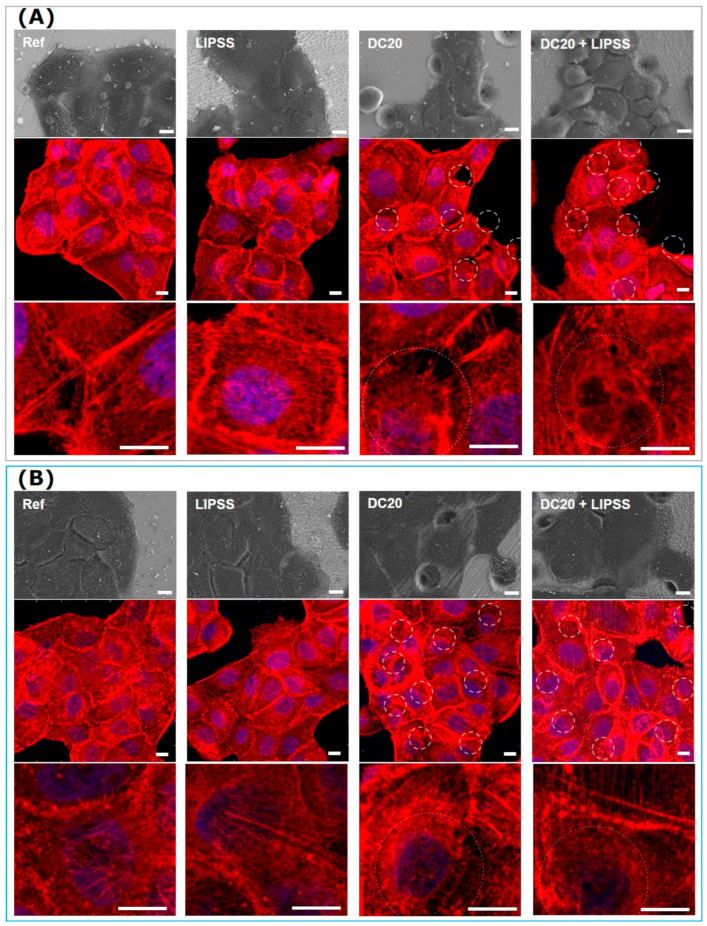
Cell morphology and actin cytoskeleton organization of human keratinocytes (HaCaT) after 24 h on different laser-structured metal specimens: (**A**) medical steel and (**B**) Ti_6_Al_4_V. Note that the cells grew on both metals and were independent of the structuring process. After 24 h, there was no apparent change in cell morphology and actin cytoskeleton. HaCaTs could span over the micro-dimple clusters and their actin filaments. (First row: FE-SEM Merlin VP compact, 1000× magnification, 5 kV, HE-SE detector. Second and third rows: LSM780, 63× oil objective, both Carl Zeiss; actin in red, nucleus in blue, underlying micro-dimples are marked as dotted circles, scale bars = 10 µm).

Physicochemical surface properties can influence cell adhesion, spreading, morphology, and actin cytoskeleton organization. Therefore, the impact of the laser structuring of metals on HaCaTs morphology was observed by imaging techniques (SEM and LSM) after 24 h. In Figure 6, the typical island formation of the keratinocytes on all specimens could be detected. Morphological differences, for example in cell size and shape, could not be observed in the FE-SEM images (Figure 6A,B, first row). Actin cytoskeleton organization also revealed no differences (Figure 6). Strong actin cortical fibers at the cell edge and thin filaments in the entire cell body were visible in the HaCaTs on all specimens. Interestingly, there was no evidence of any effect on morphology or actin formation due to the dimple microstructure; the cells grew over the dimples.

Thus, laser structuring affects only the initial process (up to 2 h), which is the advantage of HaCaTs in DC20 + LIPSS. In comparison, after 24 h, no difference in morphology was detectable.

#### 3.2.5. Cell Growth after 7 Days

**Figure 7:**
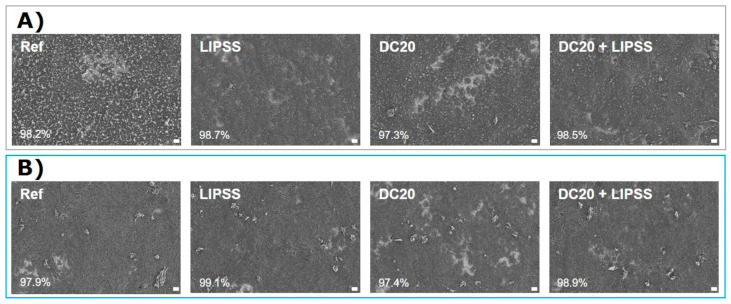
Growth of human keratinocytes (HaCaT) after 7 days on different specimens: (**A**) on steel and (**B**) on Ti_6_Al_4_V. Note that the cell density is nearly confluent. Only the cluster microstructure without LIPSS (DC20) indicated some free spaces with a confluence < 97.4%. HaCaTs could spread into and over the dimple structure and were not inhibited in extension. (FE-SEM Merlin VP compact, 1000× magnification, 5 kV, HE-SE detector scale bars = 20 µm; quantification of confluence by ImageJ).

After a 7-day HaCaT cultivation, it could be shown by FE-SEM that the cells were able to cover the surfaces nearly completely (Figure 7). A slight difference was detectable in cell growth on specimens with micro-dimple clusters made of both metal materials (DC20). Here, gaps in the cell lawn were still visible. Otherwise, the keratinocytes were able to span the cavities of the dimples. The micro-dimple and nanostructure (DC20 + LIPSS) facilitated the confluent colonization of the surface.

## 4. Discussion

The present study showed that the adhesion and growth of human keratinocytes (HaCaT) on metal surfaces (steel and Ti_6_Al_4_V) could be improved by surface modifications induced by ultrashort laser pulses. This study aimed to generate bioinspired structures (deer antlers) on external bone fixators (Schanz Pins) for the enhanced cell response—adhesion and growth—of dermal cells, so that the skin barrier can rebuild rapidly.

After implanting a biomaterial, competition between cells and bacteria occurs at the implant interface, the so-called “race for the surface” [27]. There are two strategies available to prevent implant infections: the direct route of creating antibacterial surfaces and the indirect way of creating cell-adhesive surfaces. Regarding the “race for the surface”, following the indirect route with cell-adhesive surfaces means that cells first encounter the implant surface. The cells adhere, spread, and migrate quickly within a short time. The surface is then covered with cells/tissue, and the bacteria have no “space” to colonize, eventually forming a biofilm directly on the material surface. The prerequisite for winning “the race for the surface” is the rapid initial cell adhesion we want to achieve in this study, inspired by nature, using ultrashort laser pulses.

Since Kierdorf et al. [39] undertook detailed imaging studies of deer antlers’ pedicle surface structure, interest in this surface architecture has grown. The first report about the application of the dear antler surface structure was published by Pendegrass et al. [40], who wanted to facilitate the idea of amputees using a metal rod that is anchored into the human femur, crossing the skin barrier, to attach a leg prosthesis securely. These so-called Endo–Exo prostheses are now in widespread use [41,42]; the problem of bacterial contamination has been addressed by other means, such as vicryl mesh or silver coatings [43,44,45]. Surface modifications of orthopedic implants are currently used for their tribological effects on joint endo-prostheses [34].

The study aimed to topographically modify the surfaces of percutaneous fixator pins used to treat various bone diseases, such as fractures or joint arthrosis [1,2,3,4,46,47]. Typically, these pins are made of polished metal (titanium alloy or stainless steel); therefore, skin cells do not adhere well [2,3,4,5,6,48]. To optimize the adhesion of skin cells like keratinocytes, we started by topographically structuring the Schanz Pins.

Ultrashort laser pulses could create precise and high-quality nano- and microstructures on the metal specimens [49,50]. Due to bionic specifications and the deer antler’s natural texture in the skin penetration area, the focus was on dimple clusters with additional nanostructures (LIPSS) coverage. The nano- and microstructures were adequately characterized using laser scanning microscopy and SEM analysis [34]. Although the selected microstructures of the medical steel and Ti_6_Al_4_V were almost identical and identical cleaning steps after laser texturing were also carried out, the wetting tests showed a different wetting behavior of the two sample materials. The most important factors influencing the wetting properties of laser-structured surfaces include the geometry of the microstructures, like shape, size, and arrangement, and the surface energy and purity of the surface, such as local impurities or oxide layers [49]. In addition, possible coatings and environmental conditions such as temperature and humidity also play a non-negligible role. Despite identical texturing, the different measured water contact angles on medical steel and Ti_6_Al_4_V might be primarily caused by the various sample preparations, as the steel discs were additionally polished (Sa = 0.03 µm) due to the sharp-edged EDM surface. In comparison, Ti_6_Al_4_V samples were microstructured in a finely turned state (Sa = 0.23 µm). In addition, the Ti_6_Al_4_V surfaces had a much higher tendency to form an oxide layer after laser-structuring in an air atmosphere without an inert gas environment. Titanium oxide can have a high surface energy and thus promote wetting, which can additionally explain the lower measured contact angles. Cunha et al. [51] showed that laser-induced nano-textured Ti_6_Al_4_V surfaces showed increased hydrophilicity compared to the smooth reference, with anisotropic textures exhibiting better wettability.

So, the samples made of Ti_6_Al_4_V tended to be more hydrophilic, while the structured specimens made of medical steel showed the strongest hydrophobic wetting properties after laser structuring. Surface structuring is a parameter when controlling surface wettability and cell adhesion [49,52]. Thus, biomaterial surface properties such as topography, charge, energy, and wettability can regulate cell behavior.

It has been postulated that hydrophilicity can increase the adsorption of extracellular matrix (ECM) proteins, and these ECM proteins are essential to forming a scaffold for cell adhesion [53,54]. In our study, wettability had no detectable effect on the cell behavior of keratinocytes. Improved adhesion was demonstrated for all laser structures, although micro-dimple and nanostructure DC20 + LIPSS were hydrophobic on steel (θ = 99°) and hydrophilic on titanium alloy (θ = 28°). The finding that substrate wettability did not correlate closely with cell adhesion suggests that other surface properties besides wettability play an independent role in stimulating cell behavior. Therefore, the increased cell adhesion and proliferation rate were not due to wettability, but topography. Nano- and microtopographies have been shown to influence cell responses, such as adhesion, spreading, and growth [13,16,17,20,21,52,55]. Cells can perceive and react to micro- and nanoscale topography [13,16,17,20,22,52,56]. Cells’ responses to surface topography are mediated by integrins [57] and focal adhesions [13,58], but also by their hyaluronan coat [59]. Teixeira et al. [13] also showed that epithelium aligns better with topographies with lateral dimensions from the nano- to the micronscale due to improved focal adhesion and actin cytoskeleton alignment.

Further, in vitro studies support these findings, with different cell behaviors depending on the topographic scale. Lim et al. [56] showed that nanotextured surfaces induce higher cell adhesion than flat controls. In addition, they showed improved cell adhesion to hydrophobic PLLA via nanotopographic surface fabrication [56]. The study by Oh et al. [59] also showed that the formation of nanotubes on titanium led to the improved adhesion/proliferation of osteoblasts, with the filopodia of the growing cells penetrating and anchoring into the pores of the nanotubes. The study by Oh et al. [60] also showed that the formation of nanotubes on titanium led to the improved adhesion/proliferation of osteoblasts, with the filopodia of the growing cells penetrating the pores of the nanotubes and anchoring there. Oh et al. [60] attributed the improved cell growth to the pronounced topographical property and the significantly increased surface area.

However, no conclusive results have yet been obtained on the ideal multiscale topographies, and the underlying mechanism/effects of the material on cell behavior still need to be determined [22,52].

Furthermore, studies have yet to investigate how dermal keratinocytes behave on biomaterials. The laser structuring of titanium [22,61] or zirconia [37,62] has been described sporadically, but only a few studies have addressed steel [63,64]. In addition, osseointegration for orthopedic [5,22,52] or dental [37] materials, but not for the skin barrier, is considered. To investigate the behavior of the dermal cells in relation to laser-structured materials, HaCaTs, widely used in skin biology and differentiation, were chosen [65].

Bioactivity and biocompatibility play essential roles in the success of biomaterials [66]. For example, Li et al. [66] showed in their study that using bioinspired micro- and nanostructures represented an innovative functionalization strategy to control osteointegration on titanium alloy implants.

A biomaterial should provide an environment for cells to spread and grow to ensure tissue regeneration. Cells perceive their physico-chemical environment, leading to the reorganization of focal adhesion and phenotypic changes, e.g., of the actin cytoskeleton [52]. Cell attachment is an essential initial response of cells to material surfaces [18,19,27,52,53]; therefore, the initial adhesion (10 min) of HaCaTs to laser-indued materials was addressed. Surface modification with the micro-dimple cluster (DC20) leads to improved adhesion, which was detected when combining the micro-dimple structure with additional nanostructures (DC20 + LIPSS). However, the LIPSS nanostructure alone did not affect initial cell adhesion.

The study by Cunha et al. [49] also showed that the laser nano-texturing of titanium reduced human mesenchymal stem cells’ spreading and adhesion, and thus, finally inhibited proliferation. In later studies, LIPSS-covered micro-columns were also shown to reduce hMSC spreading, but an improvement in hMSC differentiation into osteoblastic lineage was evident after a time [49]. In contrast, using femtosecond laser-textured Ti_6_Al_4_V (combination of microgrooves and oriented nanostructures), Dumas et al. [67,68] detected the formation of cytoplasmic extensions with focal contacts on the nanostructures, a higher propagation velocity on all textured surfaces compared to polished titanium, and no impairment of proliferation and osteogenic differentiation in MSC.

The spreading of the cells on the surfaces is also crucial for the adhesion of the cells to the substrate, since the cells require anchors on the substrate [69]. Regarding cell spreading (2 h), the LIPSS nanostructure showed notable improvements over the polished metals (Ref). This property was considerably enhanced with the micro-dimple clusters (DC20 + LIPSS). Interestingly, this positive effect is not seen for micro-dimples without LIPSS (DC20). This shows that the individual elements of the structures cannot explain the properties of their combination, but the interaction during spreading is essential. Therefore, the superposition principle is not applicable, and each design must be studied individually. The much smaller structured area is one factor evoked to explain why this is not evident in the individual dimples. This may animate the cells locally in small areas, but the effect is not measurable due to the small structured portions.

Due to the good initial adhesion ability, no differences in cell morphology and actin organization could be detected after 24 h of cultivation of HaCaTs. The cells could grow into micro-dimple structures (DC 20 & DC20 + LIPSS), spread over them, and settle in the transition. Also, Senthil et al. [70] showed the excellent growth of HaCaT cells on cellulose nanofiber. Cell spreading is one of the many processes that involves cytoskeletal restructuring. After 24 h, no influence of actin organization on laser structuring could be detected. Cell spreading after 7 d led to a uniform cell carpet for all surfaces. The micro-dimple clusters alone formed the most significant deviation (DC20). These free places could be closed by combination with additional LIPSS nanostructure (DC20 + LIPSS).

In addition to the actin cytoskeleton, cell adhesion structures such as hemidesmosomes and focal adhesion play a crucial role in keratinocyte’s initial adhesion and migration [71,72]. Löffek et al. [71] found in their study that the hemidesmosomal transmembrane component collagen XVII plays an essential role in epidermal anchoring by activating integrin-dependent PI3K activation and stabilizing lamellipodia at the anterior margin of re-epithelializing wounds. Also, Jacków et al. [72] showed that focal adhesions and hemidesmosomes regulate adhesive and migratory wound healing behaviors and the formation of lamellipodial protrusions in keratinocytes. Further experiments, such as on the expression of integrins or vinculin, will be necessary to understand the underlying mechanisms of improved adhesion to laser-induced structures.

Furthermore, the micro-dimple structures showed clear tendencies towards a higher metabolic turnover per cell after 24 h, and the titanium alloy structures showed a significant improvement in viability compared to the planar reference. Again, the nanostructured LIPSS showed no improvement in HaCaT proliferation. The influence of the material should also be noted here: the cells on the steel samples have a significantly higher turnover overall (approx. 50% increase compared to the titanium alloy). Cecato et al. [73] showed an increased rate of growth of gingival cells (keratinocytes and fibroblasts) on steel compared to titanium or polyether ether ketone (PEEK).

Both micro-dimple structures (DC 20 and DC20 + LIPSS) are suitable, and also show that laser structuring generally does not favor adhesion. The advantages of targeted texturing over randomized methods (etching) are apparent here. It was also demonstrable that the adhesion of HaCaT was fundamentally higher on medical steel than on titanium alloy. The overview of all laser-induced surface structures (Appendix A and Appendix B) shows that the micro-dimples and the starting material can positively and negatively affect the cell response. Therefore, the arrangement and size of the micro-dimples have a significant influence on the cell behavior. Thus, we hypothesize that the largest possible structured surfaces, with structures friendly to cells on different hierarchical scales, will be the most effective.

The present study shows that learning from nature with bionic approaches is a promising way to improve the attachment behavior of cells. The bioinspired ultrashort laser pulse structuring of metals clearly showed improvements in cell adhesion and the spreading of HaCaTs. The cells can adhere to the microstructures and ensure rapid surface coverage. In this way (fast cell adhesion and wound sealing), the growth of bacteria can be indirectly prevented, which is an essential factor to the success of the implanted pins. The antibacterial functions of material surfaces are crucial for reducing clinical infections. Studies have shown that laser-induced surface topographies, such as LIPSS, exhibit excellent antibacterial adhesion behavior compared to polished surfaces [74,75,76]. Surface micro/nanostructures reduce the contact area between materials and bacteria [73]. Furthermore, it is assumed that the surface oxidation that occurs during laser irradiation increases the surface energy of the materials, and thus additionally impairs the interactions between the bacterial membranes and the material surfaces [74,75]. Microbial analyses of our nano- and microstructures are also planned in future investigations.

In addition, we would like to investigate the underlying mechanisms further, and for this purpose, we want to test other mechanical properties, such as surface charge, in following studies. In addition to characterizing and validating the biomaterial’s physicochemical properties, understanding the cell’s molecular mechanisms is crucial for optimizing the parameters in tissue regeneration. To further investigate the healing of skin wounds, we should include in the following analyses the expression of genes involved in glycolysis, oxidative phosphorylation, pentose phosphate shunts, and glutamine anaplerosis [77].

The heuristic approach remains the best tool of choice, since no superposition principle is applicable, and different cell types prefer other structures based on factors such as average cell size.

## 5. Conclusions

**Figure 8:**
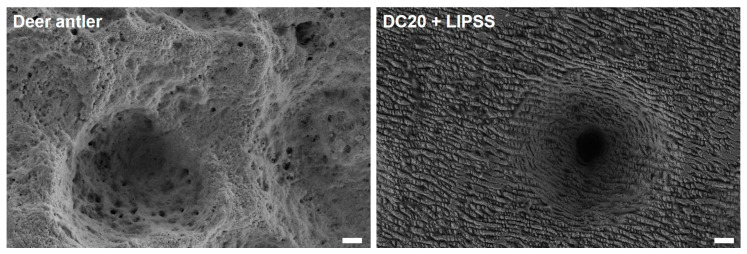
Natural versus artificial structures. Comparison of nano- and microstructures of a deer antler surface (**left**) and a steel surface with laser-induced micro-dimple clusters combined with nanostructures from periodic surface structuring (DC20 + LIPSS) (**right**). This laser structure is similar to nature’s model and facilitates the best effects on initial human keratinocyte adhesion. (FE-SEM Merlin VP compact, 5 kV, HE-SE detector; 3000× magnification, scale bars = 2 µm).

Intending to improve metallic surfaces for external bone fixators made from medical steel and titanium alloy Ti_6_Al_4_V, this study presents an ultrashort laser pulse structure inspired by deer antlers, with their specific nano- and microstructure. We identified the best surface topography compared to the antler’s micro- and nanostructure by combining micro-dimple clusters and laser-induced periodic surface structures (DC20 + LIPSS) (Figure 8). These prepared surfaces showed optimal results for the initial adhesion and spreading of keratinocytes HaCaT compared to polished references, and also to the nanostructured LIPSS alone. Moreover, it was seen that the combination of nano- and microstructures is more suitable for cell adhesion processes than either topographical size range by itself.

Laser processing as a technology can produce biofunctional surfaces in a precise and controlled manner to achieve the desired topographical aspects of biomaterials and biological response, such as rapid cell adhesion and fast surface occupation by cells.

## Figures and Tables

**Table 1:** Structural characteristics of medical steel and Ti_6_Al_4_V samples (structure type; dimple diameter, depth, and texture density).

Structure	Ø Dimple (µm)	Depth (µm)	Dimples per mm^2^
**Ref** (polished)	-	-	-
**LIPSS** (nanostructure)	-	-	-
**DC20** (micro-dimple cluster)	20	10	80
**DC20 + LIPSS** (micro-dimple + nanostructure)	20	10	80

Note that the deer antler’s microstructure displayed 26.3 ± 1.9 dimples per mm^2^ with a diameter of 41.9 ± 2.4 µm (n = 5 with 3 sections; mean ± s.e.m.).

**Table 2:** Water contact angle on different medical steel and Ti_6_Al_4_V samples.

Structure	Contact Angle, θ [°]
**Ref** (polished)	58.9 (steel) 80.6 (Ti_6_Al_4_V)
**LIPSS** (nanostructure)	66.7 (steel) 8.5 (Ti_6_Al_4_V)
**DC20** (micro-dimple cluster)	62.9 (steel) 40.5 (Ti_6_Al_4_V)
**DC20 + LIPSS ** (micro-dimple + nanostructure)	98.7 (steel) 28.2 (Ti_6_Al_4_V)

**Figure A1:**
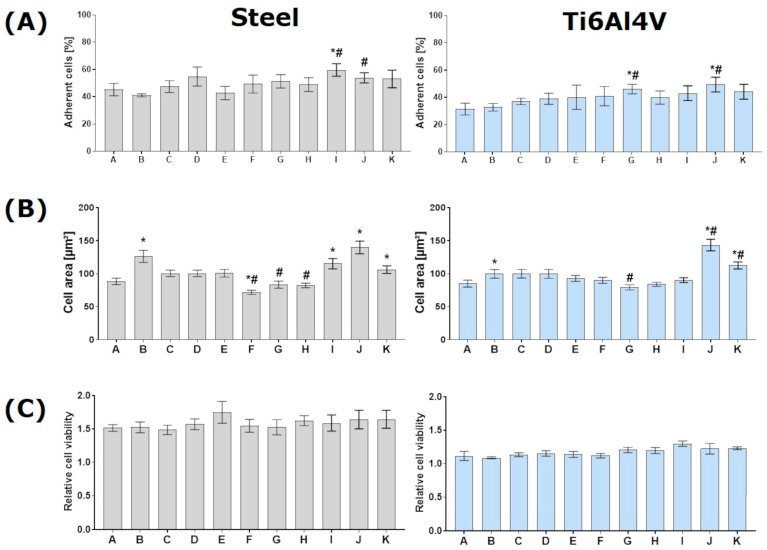
Cellular responses of human keratinocytes (HaCaT) on different specimens within 24 h: (**left**) on medical steel and (**right**) on titanium alloy (Ti_6_Al_4_V). Note that specimens A-Ref, B-LIPSS, H-DC20, and J-DC20 + LIPSS, respectively, were presented in the article. (**A**) Results regarding initial adhesion after 10 min show that only J (DC20 + LIPSS) could increase cellular attachment on both materials (FACSCalibur; n = 4, mean ± s.e.m.). (**B**) The spreading capacity of HaCaTs on structure J (DC20 + LIPSS) was most pronounced. The cell area was significantly improved on nanostructure B (LIPSS) and another nano- and microtopography combination (K). However, these structures did not show a significant increase in initial adhesion. It should be noted here that laser structures both promote and inhibit cell spreading (ImageJ; n = 40 cells of SEM images, mean ± s.e.m.). (**C**) Viability data show no effects on metabolic activity. However, the lower viability of HaCaT on Ti6Al4V than medical steel should be noted (Anthos reader; n = 4, mean ± s.e.m). (Statistics: adjusted Mann–Whitney test: * *p* ≤ 0.05 to Ref, # *p* ≤ 0.05 to LIPSS.)

## Data Availability

The data sets generated and/or analyzed during the current study are available from the corresponding author on reasonable request. The data set is stored on the local Rostock University Medical Center (UMR) server. The data sets supporting the in vitro investigations of this article are available in the zenodo repository: https://doi.org/10.5281/zenodo.10148655 (accessed on 1 January 2024).

## Appendix A

**Table A1:** Characterization of all different nano- and microstructures used in the project. Illustration of materials (LSM, VK-X200, Keyence; scale bars = 50 µm; SEM EVO 15, Carl Zeiss; scale bars = 10 µm).

Structure	LSM	Ø Dimple (µm)	Depth (µm)	Dimples per mm^2^	Contact Angle (°)
A (polished reference, **Ref**)		-	-	-	58.9 (steel) 80.6 (Ti_6_Al_4_V)
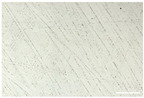
B (nanostructure, **LIPSS**)		-	-	-	66.7 (steel) 8.5 (TiAl_4_V_5_)
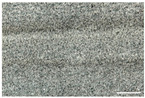
C (microstructure, periodic dimples, 42/mm^2^)		50	20	42	60.9 (steel) 62.2 (Ti_6_Al_4_V)
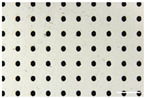
D (microstructure, periodic dimples, 81/mm^2^)		50	20	81	68.9 (steel) 43.8 (Ti_6_Al_4_V)
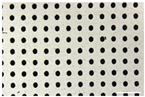
E (micro- and nanostructure, periodic dimples (42/mm^2^) + LIPSS)		50	20	42	71.55 (steel) 22.2 (Ti_6_Al_4_V)
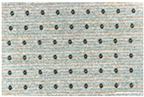
F (micro- and nanostructure, periodic dimples (81/mm^2^) + LIPSS)		50	20	81	78.9 (steel) 42.5 (Ti_6_Al_4_V)
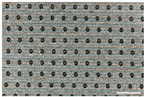
G (micro-dimple cluster, Ø 35µm)		35	10	80	72.8 (steel) 40.8 (Ti_6_Al_4_V)
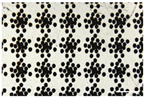
H (microstructure, dimple cluster, Ø 20 µm; **DC20**)		20	10	80	62.9 (steel) 40.5 (Ti_6_Al_4_V)
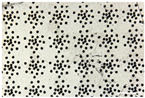
I (microstructure, dimple cluster (Ø 35 µm) + LIPSS)		35	10	80	94.6 (steel) 28.9 (Ti_6_Al_4_V)
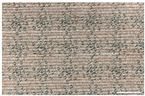
J (microstructure, periodic dimples (Ø 20 µm) + LIPSS, **DC20 + LIPSS**)		20	10	80	98.7 (steel) 28.2 (Ti_6_Al_4_V)
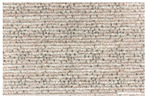
K (microstructure, periodic dimples (400/mm^2^) + LIPSS)		50	10	400	87.8 (steel) 26.4 (Ti_6_Al_4_V)
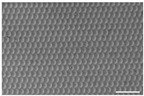

## Appendix B

Results of the in vitro investigation with all different nano- and microstructures in the project.
